# Pharmacological inhibition of *O*-GlcNAcase (OGA) prevents cognitive decline and amyloid plaque formation in bigenic tau/APP mutant mice

**DOI:** 10.1186/1750-1326-9-42

**Published:** 2014-10-26

**Authors:** Scott A Yuzwa, Xiaoyang Shan, Bryan A Jones, Gang Zhao, Melissa L Woodward, Xiaojing Li, Yanping Zhu, Ernest J McEachern, Michael A Silverman, Neil V Watson, Cheng-Xin Gong, David J Vocadlo

**Affiliations:** Department of Molecular Biology and Biochemistry, Simon Fraser University, 8888 University Dr, Burnaby, BC V5A 1S6 Canada; Department of Neurosciences and Mental Health, The Hospital for Sick Children, Toronto, ON M5G 0A4 Canada; Department of Psychology, Simon Fraser University, 8888 University Dr, Burnaby, BC V5A 1S6 Canada; Department of Neurochemistry, New York State Institute for Basic Research in Developmental Disabilities, 1050 Forest Hill Road, Staten Island, NY 10314 USA; Department of Biological Sciences, Simon Fraser University, 8888 University Drive, Burnaby, British Columbia, V5A 1S6 Canada; Department of Chemistry, Simon Fraser University, 8888 University Dr, Burnaby, BC V5A 1S6 Canada; Alectos Therapeutics Inc, 8999 Nelson Way, Burnaby, BC V5A 4B5 Canada

**Keywords:** tau, Amyloid precursor protein, *O*-GlcNAc, Thiamet-G

## Abstract

**Background:**

Amyloid plaques and neurofibrillary tangles (NFTs) are the defining pathological hallmarks of Alzheimer’s disease (AD). Increasing the quantity of the *O*-linked N-acetylglucosamine (*O*-GlcNAc) post-translational modification of nuclear and cytoplasmic proteins slows neurodegeneration and blocks the formation of NFTs in a tauopathy mouse model. It remains unknown, however, if *O*-GlcNAc can influence the formation of amyloid plaques in the presence of tau pathology.

**Results:**

We treated double transgenic TAPP mice, which express both mutant human tau and amyloid precursor protein (APP), with a highly selective orally bioavailable inhibitor of the enzyme responsible for removing *O*-GlcNAc (OGA) to increase *O*-GlcNAc in the brain. We find that increased *O*-GlcNAc levels block cognitive decline in the TAPP mice and this effect parallels decreased β-amyloid peptide levels and decreased levels of amyloid plaques.

**Conclusions:**

This study indicates that increased *O*-GlcNAc can influence β-amyloid pathology in the presence of tau pathology. The findings provide good support for OGA as a promising therapeutic target to alter disease progression in Alzheimer disease.

## Background

The formation of oligomers and aggregates of β-amyloid peptides derived from amyloid precursor protein (APP) is a causative early factor giving rise to Alzheimer Disease (AD) [1–3]. Neuritic plaques are the pathological feature associated with deposition of large aggregates of β-amyloid peptides. Mutations in the human APP gene can give rise to autosomal dominant early-onset forms of AD, which resemble late-onset AD (hereafter simply AD) both clinically and at the histopathological level [4–6]. Furthermore, some mutations in APP also confer protection against AD [7]. The deleterious APP mutations driving early-onset AD appear sufficient to promote, within humans, the formation of the other classical pathological hallmark of AD known as the neurofibrillary tangles (NFTs). NFTs are formed as a result of the self assembly of hyperphosphorylated microtubule-associated protein tau into paired helical filaments (PHFs) which then aggregate into larger structures known as NFTs [8–11]. Human mutations in the tau gene (*MAPT*), on their own, can also give rise to a group of neurodegenerative diseases referred to as frontotemporal dementia linked to chromosome-17 (FTDP-17), one group of a number of different types of tauopathy [12, 13]. However, human *MAPT* mutations do not cause the formation of neuritic plaques, thereby lending significant support for β-amyloid peptide formation as a factor upstream of tau in AD.

Studying the relationship between NFTs and neuritic plaques in AD is complicated by the fact that rodent models that carry human APP mutations do not generally display detectable NFT pathology [14, 15], even in the presence of neuritic plaques and cognitive impairment. Conversely, mouse models that express human *MAPT* mutations, such as the P301L mutant expressing JNPL3 mice, do not produce neuritic plaques [16]. For this reason several groups have developed mouse models of AD that recapitulate both pathological features of AD. One model, generated by Lewis *et al*., referred to as the TAPP mouse, resulted from crosses of JNPL3 tau mice with mice expressing the most common APP mutant (Swedish mutation K670N/M671L, APPSwe; Tg2576) [17]. Differing from the parent single transgenic models, TAPP mice brains contain both plaques and NFTs. Notably, these TAPP mice display enhanced tau pathology as compared to JNPL3 mice, suggesting that β-amyloid peptides accelerate the formation of NFTs [17], a proposal supported by other studies involving intracerebral injection of β-amyloid Aβ42 fibrils into P301L tau transgenic mice [18]. The TAPP mouse model is therefore well suited to study therapeutic strategies that might impact neuritic plaques or NFT formation in a setting that captures these two synergistic pathologies.

Previously, we reported a potential disease modifying approach aimed at reducing toxicity associated with tau aggregation and NFT formation. This approach involved increasing global levels of a little-studied post-translational modification known as the *O*-GlcNAc modification [19]. Chronic increases in the levels of *O*-GlcNAc modification in JNPL3 mouse brains and spinal cords slowed neuronal loss and reduced the number of NFTs formed over a treatment period of several months [20]. *O*-GlcNAc modification of proteins involves the attachment of single *N*-acetyl-D-glucosamine residues to the hydroxyl side chains of serine and threonine residues of proteins [19]. The *O*-GlcNAc modification differs from classical forms of glycosylation found on the outside of the cell and within the secretory pathway because *O*-GlcNAc is found in the nucleocytoplasm and it does not have additional sugar residues attached to it to form more complex structures [19]. The fact that *O*-GlcNAc can be added or removed from a particular protein multiple times during the lifespan of the protein makes it a dynamic modification [21] somewhat akin to protein phosphorylation, which is likewise reversible. Installation of *O*-GlcNAc on serine or threonine is carried out by a single glycosyltransferase referred to as *O*-GlcNAc transferase (OGT), which uses the high-energy donor sugar uridine 5’-diphospho-*N*-acetyl-D-glucosamine (UDP-GlcNAc) as its substrate [22, 23]. A single glycoside hydrolase, *O*-GlcNAcase (OGA), is tasked with the hydrolytic cleavage of *O*-GlcNAc from modified proteins [24, 25]. To increase global *O*-GlcNAc levels in JNPL3 mice we made use of a potent (*K*_i_ =21 nM) and selective (37000-fold for human OGA over functionally related human β-hexosamindases) inhibitor of OGA referred to as Thiamet-G, which blocks removal of *O*-GlcNAc from modified proteins [26]. Thus, even as OGA is inhibited, OGT can continue adding *O*-GlcNAc onto to modified proteins resulting in elevated *O*-GlcNAc within cells [26, 27].

We previously showed that increasing *O*-GlcNAc levels has beneficial effects in the JNPL3 mouse model of tauopathy [20]. Recently APP has been found to be *O*-GlcNAc modified and one study suggested that *O*-GlcNAc might alter β-amyloid production by regulating APP processing [28, 29]. To study the role of *O*-GlcNAc on APP and β-amyloid production in mice exhibiting both tau and β-amyloid pathologies, we undertook a long-term study using Thiamet-G to increase the global levels of *O*-GlcNAc in TAPP mice. Here we show that Thiamet-G can increase *O*-GlcNAc levels in the TAPP mouse brain, leading to reductions in levels of both neuritic plaques and amyloidogenic β-amyloid peptides. Consistent with these findings we also find that Thiamet-G treatment blocks the onset of cognitive impairment in these animals. We also show, using cell models of β-amyloid peptide formation, that Thiamet-G does not alter the release of β-amyloid peptides from cells, suggesting that increased *O*-GlcNAc levels in mice mediate protection against β-amyloid peptides through a mechanism that is likely independent of Aβ42 release.

## Results

### Thiamet-G treatment improves performance in the Morris water maze (MWM)

**Figure 1:**
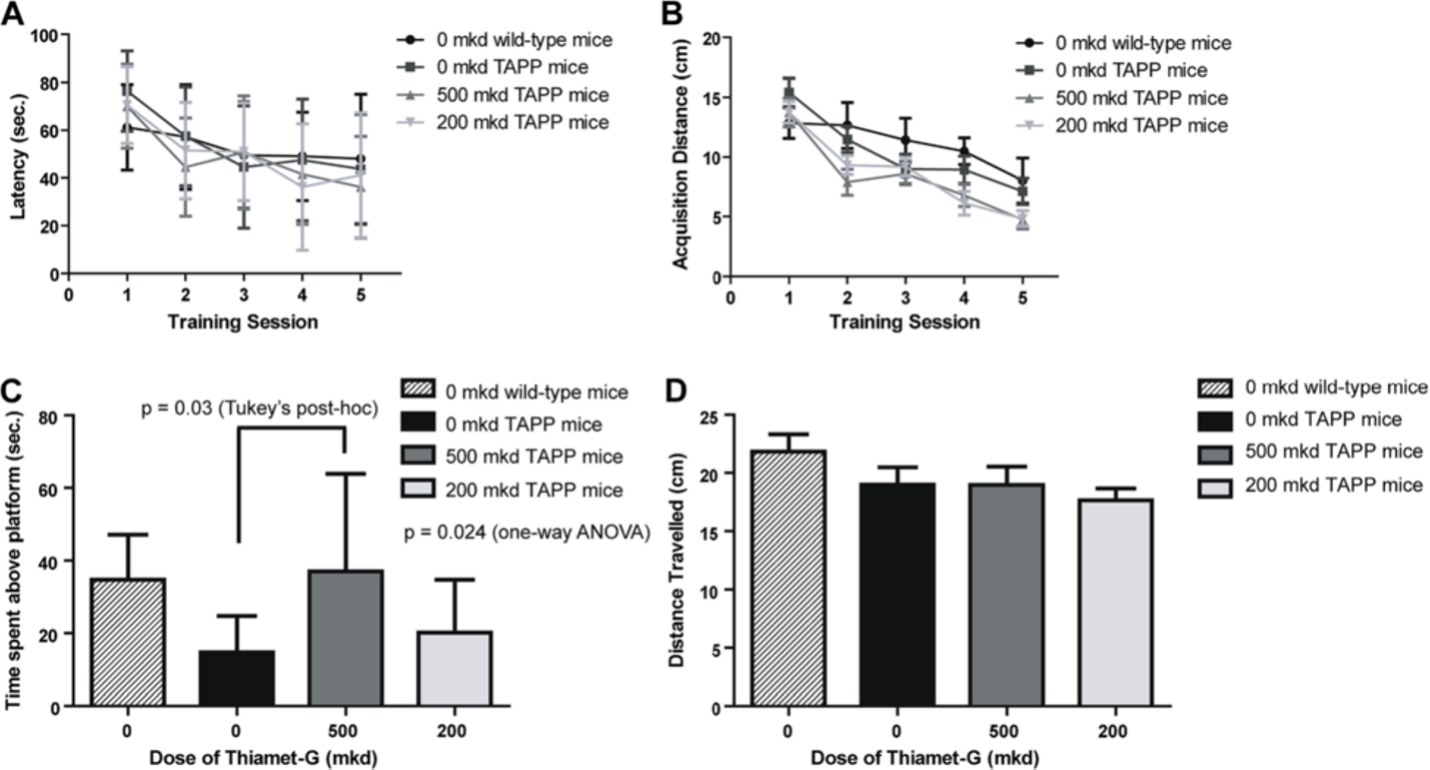
**Thiamet-G prevents cognitive decline in the TAPP mice. A**, **B**. Beginning at 30-32 weeks of age 0, 200 or 500 mkd Thiamet-G treated TAPP mice were tested for cognitive performance in the Morris water maze (MWM). Learning curves were recorded during five consecutive days of training. No significant difference was observed between any of the groups in latency to solve the maze **(A)** and while a significant main effect was noted by the ANOVA for distance travelled, the post-hoc Tukey’s analysis revealed that there were no significant differences between groups. **(B)**. **C**. During the probe trial, the latency to solve the maze was recorded and the control TAPP mice (0 mkd Thiamet-G) show significant cognitive impairment compared to untreated age-matched wild-type mice. Conversely, 500 mkd resulted in better performance than the 0 mkd Thiamet-G treated TAPP mice while the performance of 200 mkd TAPP mice is indistinguishable from the untreated age-matched wild-type mice. **D**. No differences were observed in the distance travelled during the probe trial. Error bars represent standard deviation (± S.D) and p-value result from a one-way analysis of variance (ANOVA) For all panels, n =8 for 0 mkd wild-type mice, n =17 for 0 mkd TAPP mice, n =17 for 500 mkd TAPP mice and n =19 for 200 mkd TAPP mice.

To assess whether increased *O*-GlcNAc can influence amyloid deposition or cognitive impairment in bigenic TAPP mice, we divided 60 double transgenic TAPP mice into three groups (n = 20) receiving either 0, 200, or 500 mkd of Thiamet-G in their drinking water. We have previously shown oral Thiamet-G treatment of mice over a period of months leads to sustained *O*-GlcNAc increases in the brains of mice [20]. Parental transgenic Tg2576 mice, which harbor only the APPSwe mutation, develop pronounced memory impairment starting at 6 months of age [15, 30] as judged by performance in the Morris water maze (MWM). Therefore to determine whether increased *O*-GlcNAc can influence disease progression in TAPP mice we started dosing with Thiamet-G at 10-13 weeks of age and continued until 44-47 weeks of age. Effects of OGA inhibition on cognitive impairment were assessed by performing MWM testing on each of the animals in the three groups starting between 28-32 weeks of age. All data acquisition and data entry was performed blinded with the coding being held by non-experimenters. We find that in the acquisition phase, during which animals engage in spatial learning, the latency to solve the maze was not significantly different between the groups as judged by RM-ANOVA (F5,78 = 0.668, p =0.649) (Figure 1A). Differences in the distance traveled within the maze during this learning phase were significantly different (F5,78 = 2.389, p =0.045), though a Tukey’s *post-hoc* analysis did not reveal any specific differences between groups (Figure 1B). To clarify whether behavioural changes that might be observed in these studies are due to cognition *per se* and not due to motor differences between groups*,* we examined the time spent in the outer ring as a measure of anxiety and swim speed during these acquisition trials. The swim speed was significantly different between groups (F5,78 = 2.817, p =0.022), with the Tukey’s *post-hoc* analysis indicating that the 500 mkd Thiamet-G treated group was faster than the untreated wild-type control group (p =0.042), perhaps because treatment protects against neurodegeneration of motor neurons as previously observed in JNPL3 mice [16]. There was no difference between the groups in the time spent in the outer ring (F5,78 = 0.758, p =0.582) indicating the animals showed no apparent anxiety effects.

During the probe trials, where memories formed during the acquisition phase are tested, the 500 mkd Thiamet-G group spent significantly more time over the original platform location (F5,57 = 2.835, p =0.024) than did the untreated TAPP mice (p =0.03, Tukey’s *post-hoc* analysis) and the same amount of time as compared to wild-type control mice (Figure 1C), indicating increased *O*-GlcNAc blocks cognitive impairment in TAPP mice. Because the goal of the probe trial is for the animals to locate and remain within a very small area within the pool (~25 cm^2^), the differences between the 500 mkd Thiamet-G and the untreated TAPP mice are unlikely to result from motor differences between these groups. Consistent with this view, we did not observe any difference in the distance travelled during the probe trial (Figure 1D). These data indicate treatment with Thiamet-G resulted in cognitive enhancement compared to untreated animals, to a level matching that of wild-type animals.

### Thiamet-G increases O-GlcNAc but does not impact tau phosphorylation

**Figure 2:**
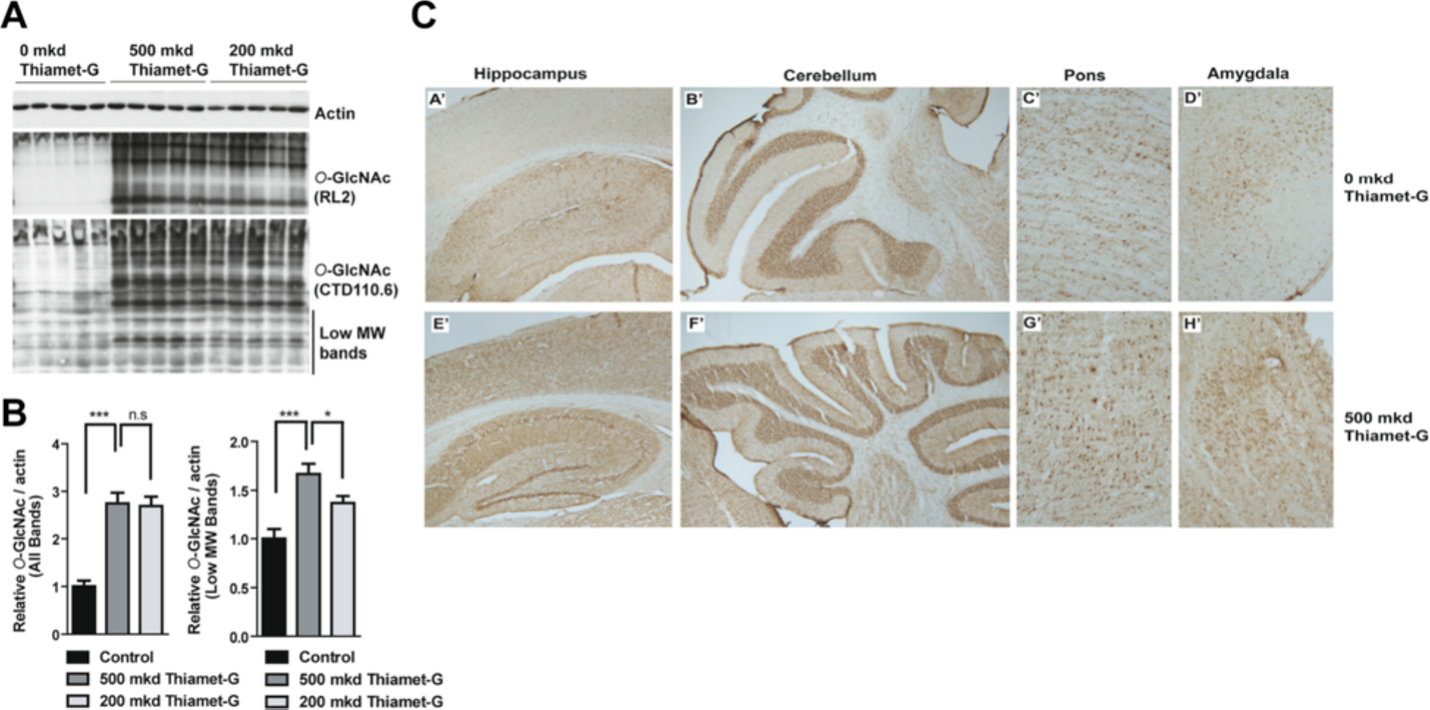
***O*** **-GlcNAc levels are increased in the TAPP mouse brain. A**. Western blots of total brain homogenates from 0, 200 and 500 mkd Thiamet-G treated TAPP mice reveals that *O*-GlcNAc levels are vastly increased (RL2 and CTD110.6) while actin indicates equal protein loading. **B**. Quantification of *O*-GlcNAc immunoreactivity (CTD110.6) normalized to actin by densitometry of all bands (left panel) or only the low molecular weight (MW) bands (bands <50 kDa, right panel). N =10 in each group. *indicates p <0.05, ***indicates p <0.001, unpaired two-tailed *t*-test) **C**. Immunohistochemical (IHC) analysis of 0 and 500 mkd Thiamet-G treated TAPP mice brain tissue reveals that *O*-GlcNAc levels are increased in all of the hippocampus **(A’, E’)**, cerebellum **(B’, F’)**, pons **(C’, G’)** and the amygdala **(D’** **, H’)**.

**Figure 3:**
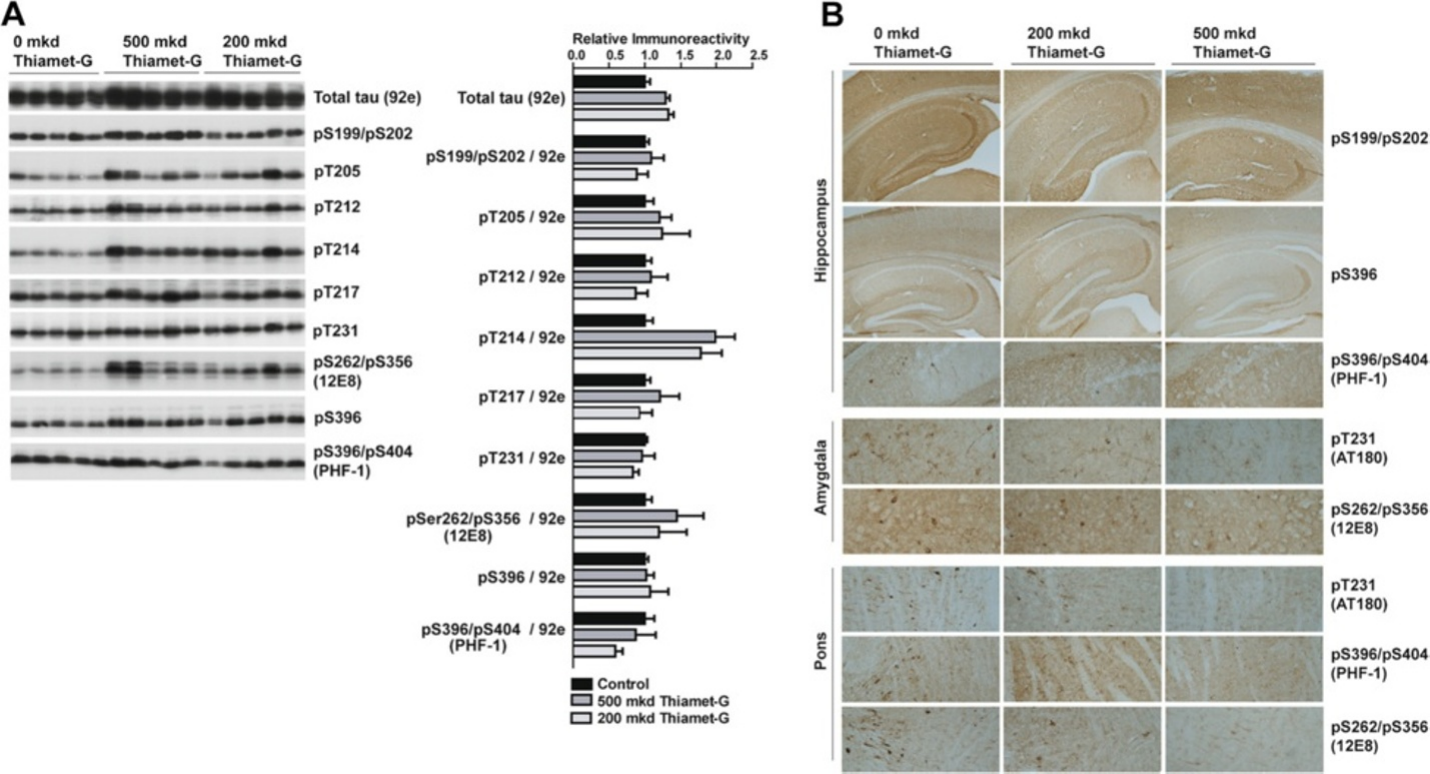
**Thiamet-G does not alter total tau or phospho-tau levels in the TAPP mice. A**. Total tau (92e) blots indicate that levels of total tau are not altered by Thiamet-G treatment. Western blots using phosphorylation state sensitive tau antibodies, pS199/pS202, pT205, pT212, pT214, pT217, pT231, pS262/pS356, pS396, and pS396/pS404 indicate that levels of phospho-tau are also not significantly altered. Quantification by densitometry of each total or phospho-tau epitope is shown to the right. Phospho-tau immunoreactivity is normalized to total tau (92e) in each case. N =10 in each group. **B**. IHC analysis using phosphorylation state sensitive tau antibodies, pS199/pS202, pS396, pS396/pS404, pT231, pS262/pS356 indicate that levels of phospho-tau are also not significantly altered in the hippocampus, the amygdala, or the pons in either of the 200 or 500 mkd Thiamet-G treated TAPP mice groups. Error bars represent standard error of the mean (± S.E.M).

**Figure 4:**
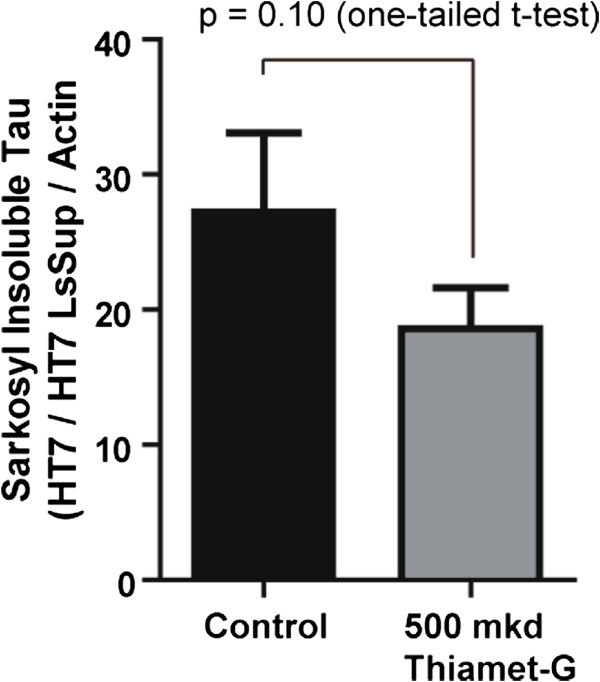
**500 mkd Thiamet-G may reduce sarkosyl-insoluble tau in the TAPP mice.** Levels of sarkosyl-insoluble tau were assessed by Western blot analysis in both the 0 and 500 mkd Thiamet-G treated TAPP mice groups and indicated a strong trend towards less sarkosyl-insoluble tau in the 500 mkd group. Error bars represent standard error of the mean (± S.E.M) and p-values result from a student’s unpaired one-tailed *t*-test. N =18 (Control) and n =17 (500 mkd Thiamet-G).

To better understand how Thiamet-G prevents impairment of cognitive performance, we first verified that *O*-GlcNAc levels were increased in treated animals (Figure 2). Immunoblotting with *O*-GlcNAc antibodies CTD110.6 and RL2 revealed dramatically increased *O*-GlcNAc levels in both the 200 and 500 mkd treated TAPP mice compared to the 0 mkd control TAPP mice (Figure 2A). We performed densitometric quantification of the *O*-GlcNAc immunoreactivity of the CTD110.6 antibody based on either all of the immunoreactive bands or only the low molecular weight (<50 kDa) immunoreactive bands (Figure 2B). This analysis revealed a trend toward lower *O*-GlcNAc levels in the 200 mkd TAPP mice group compared to the 500 mkd TAPP mice group when considering all of the immunoreactive bands. When only the low molecular weight bands are considered, *O*-GlcNAc levels were roughly 20% lower in the 200 mkd TAPP mice (p <0.05). These analyses reveal that there is a dose dependent effect of Thiamet-G on the levels of *O*-GlcNAc. We also performed immunohistochemistry (IHC) using the *O*-GlcNAc antibodies (CTD110.6 and RL2) on animals from the 0 and 500 mkd Thiamet-G treatment groups. The hippocampus and the cerebelleum have been previously shown to have high levels of expression of both OGA and OGT [31, 32] and *O*-GlcNAc levels in these structures, along with the pons and the amygdala, were particularly increased in the 500 mkd Thiamet-G treatment group (Figure 2C). Previously, we have shown that Thiamet-G does not block tau hyperphosphorylation in the transgenic parental JNPL3 mouse model [20]. We confirmed that this was also the case in the double transgenic TAPP mice by immunoblot (Figure 3A) and IHC (Figure 3B) analyses using various phosphorylation state-specific tau antibodies. Even though dramatically increased *O*-GlcNAc levels were observed (Figure 2) immunoblot analyses revealed that Thiamet-G treatment actually slightly increased the total amount of tau (92e) and the extent to which tau was phosphorylated but this did not reach statistical significance. In our previous study of JNPL3 mice [20], we found that Thiamet-G treatment reduced the amount of sarkosyl insoluble tau, which is known to correlate with the amount of fibrillar tau in 9 month old mice JNPL3 mice [33]. In a blinded experiment we therefore probed whether Thiamet-G can also reduce the amount of sarkosyl insoluble tau in the double transgenic TAPP mice. We observed a strong trend of less sarkosyl insoluble tau in the 500 mkd Thiamet-G treatment group (32% reduction), which was consistent in magnitude with our earlier JNPL3 study, although this difference did not reach statistical significance in the TAPP mice (Figure 4).

### Thiamet-G treatment reduces the number of amyloid plaques and reduces Aβ levels

**Figure 5:**
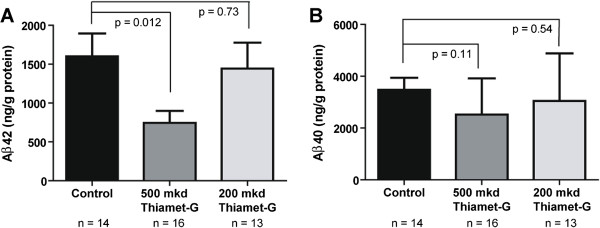
**Aβ** **40 and Aβ** **42 levels.** ELISA assays for Aβ40 and Aβ42 were used to assess the quantity of each of these species in the 0, 200 or 500 mkd Thiamet-G treated TAPP mice groups. **A**. 500 mkd was sufficient to reduce the amount of Aβ42 by half while 200 mkd was ineffective. **B**. 500 mkd Thiamet-G also showed a trend toward less Aβ40 which was not evident in the 200 mkd group. Error bars represent standard error of the mean (± S.E.M) and p-values result from student’s unpaired two-tailed t-tests For all panels, N =14 for 0 mkd TAPP mice, N =16 for 500 mkd TAPP mice and N =13 for 200 mkd TAPP mice.

Previous work has shown that at 7-8 months of age the single transgenic JNPL3 mice generally perform no worse than age matched wild-type control animals in the MWM [34]. For this reason, and the absence of significant effects on tau phosphorylation, we felt that the cognitive effects of OGA inhibition in this TAPP model reflected in the MWM results may stem from effects of Thiamet-G treatment on accumulation of β-amyloid peptides and be reflected in the extent of amyloid plaque formation. To address this possibility we performed a number of experiments in which the experimenter was blinded using coded samples. The first study established whether Thiamet-G had any impact on the quantities of the amyloidogenic forms of β-amyloid 1-40 and 1-42 (Aβ40, Aβ42). Using widely used commercially available ELISA assays for Aβ40 and Aβ42, we determined that administration of 500 mkd Thiamet-G significantly reduced the quantity of Aβ42 while the 200 mkd treatment had no significant effect on this measure (Figure 5). We also observed a trend toward less Aβ40 in the 500 mkd treatment group that was not present in the 200 mkd group (Figure 5). As described above, *O*-GlcNAc levels in the 200 mkd group are slightly lower than the 500 mkd group and thus may indicate that a sustained increase in *O*-GlcNAc levels above a certain threshold must be reached in order to influence the levels of Aβ peptides within brain.

**Figure 6:**
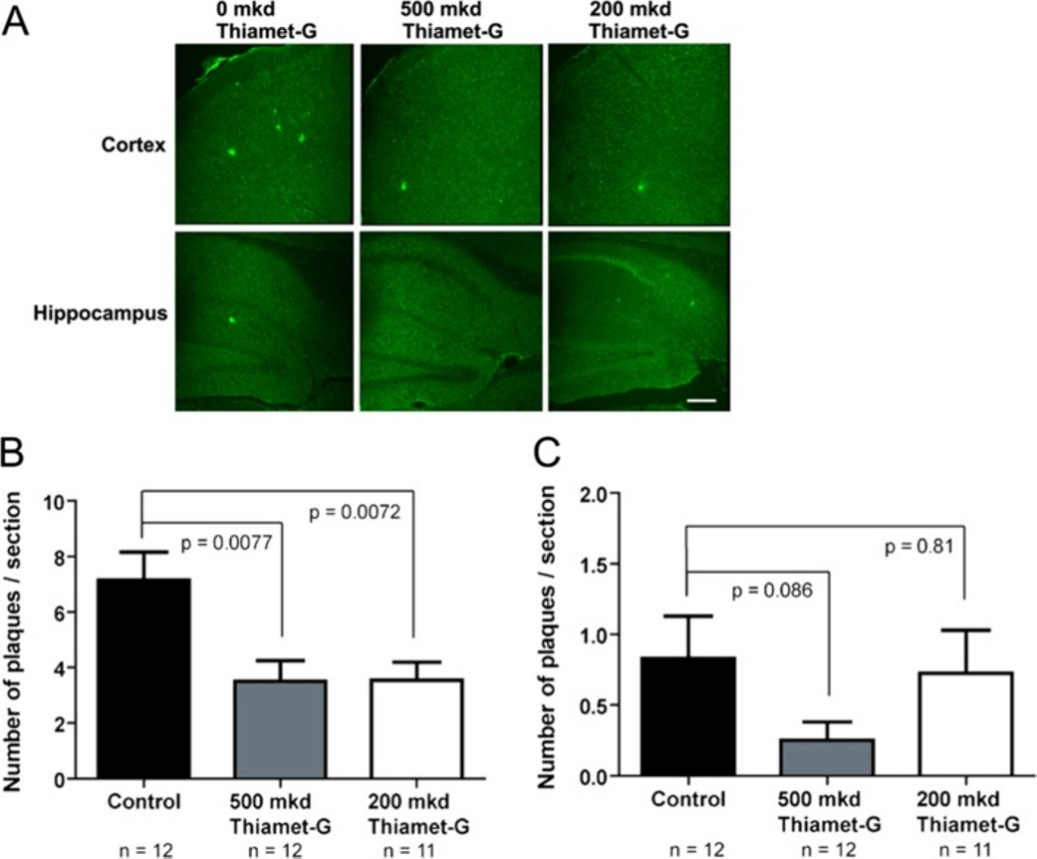
**Thiamet-G reduces plaque load in the TAPP mice. A**. Representative 6E10 immunohistochemical analysis used to assess amyloid plaque load in both the cortical and hippocampal regions. **B**. Quantitative assessment of 6E10 IHC analysis reveals that both 200 and 500 mkd Thiamet-G is sufficient to reduce the number of amyloid plaques in the cortex. **C**. 500 mkd Thiamet-G significantly reduced the number of amyloid plaques in the hippocampus whereas 200 mkd Thiamet-G was ineffective. Error bars represent standard error of the mean (± S.E.M) and p-values result from student’s unpaired two-tailed t-tests. For all panels, N =12 in each group.

Finally, we evaluated whether the reduced Aβ42 levels correlated with fewer amyloid plaques within these animals. By quantitative analysis, we observed fewer amyloid plaques in both cortical and hippocampal regions of the brain in the 500 mkd Thiamet-G treated group (Figure 6). Interestingly however, in the cortex we found that 200 mkd was sufficient to reduce the number of amyloid plaques to the same level as seen in mice treated with 500 mkd Thiamet-G despite this dose being unable to significantly reduce levels of Aβ42 as assessed by ELISA (Figure 6B). It is interesting to note that *O*-GlcNAc levels are generally higher in the hippocampus (Figure 2C) than in the cortex, yet we observe larger reductions in the number of plaques in cortex in both dose groups than observed in the hippocampus. Perhaps different brain regions have differing capacities to increase *O*-GlcNAc levels and this may be coupled to tissue dependent differences in amyloid deposition that depend on *O*-GlcNAc levels. Further, perhaps differences in the levels of Aβ42 within certain brain regions are also present yet were not detected here because the ELISAs are conducted on homogenates obtained from complete brain hemispheres. Using whole brain homogenates might mask small differences in Aβ42 levels present in certain brain regions. These observations suggest that increased *O*-GlcNAc could influence both β-amyloid peptide production or clearance as well as assembly/clearance of amyloid plaques arising from Aβ42.

### Thiamet-G treatment does not influence release of Aβ42 from cells

**Figure 7:**
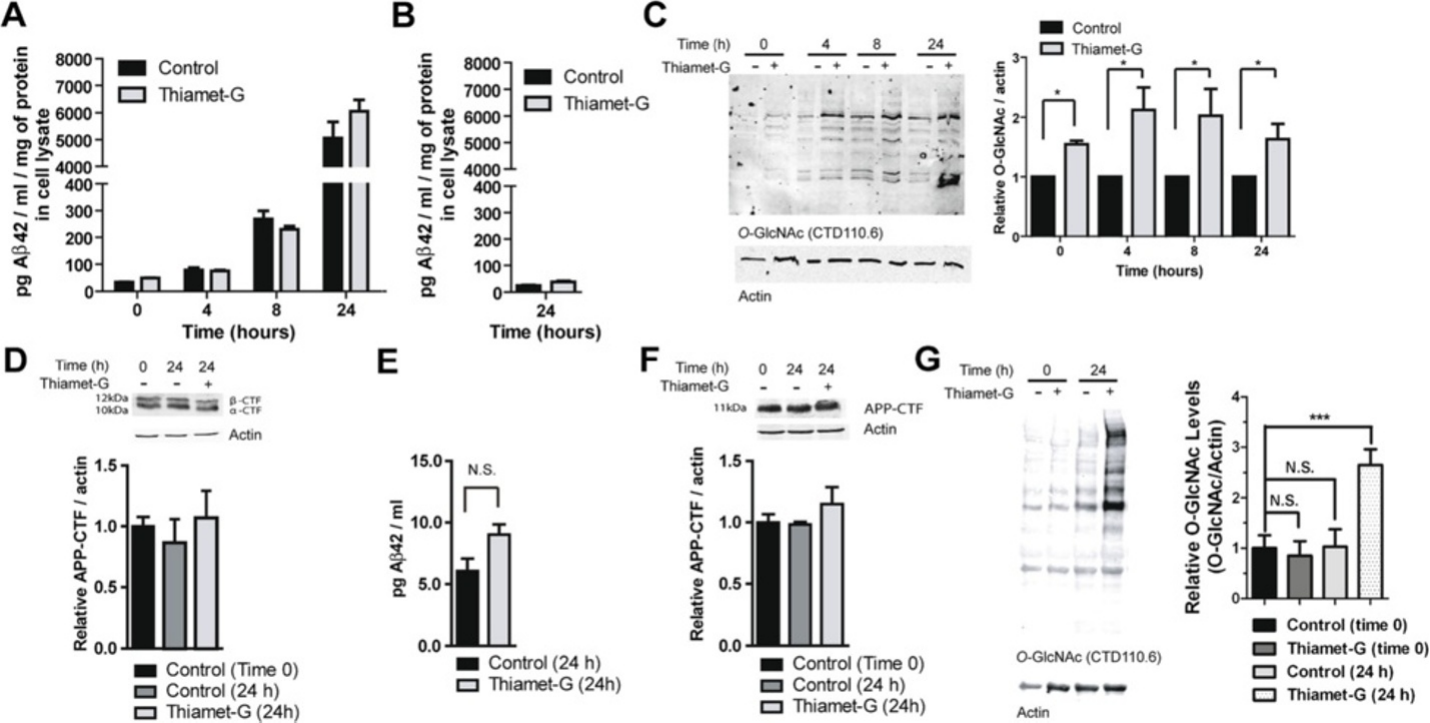
**Thiamet-G treatment of APPSwe-expressing 20E2 cells and primary hippocampal neurons does not alter APP processing. A**. 20E2 cells were treated with Thiamet-G for 16 hours, and then the amount of Aβ42 in the cell culture media, collected at the indicated time points after the replacement of new culture media, was assessed using an ELISA assay and corrected for the amount of protein in the cell lysates from each condition. This analysis reveals that Thiamet-G does not alter the amount of Aβ42 that is produced. **B**. Little Aβ42 is detected in untransfected human embryonic kidney (HEK) cells indicating that validity of this culture model. **C**. *O*-GlcNAc levels in the 20E2 cells are increased at all time points analyzed (*indicates p <0.05, paired two-tailed *t*-test). **D**. Levels of APP-CTF were unchanged in 20E2 cells treated with Thiamet-G for 24 hours. **E**. Aβ42 production is not altered in primary hippocampal neurons treated with Thiamet-G for 24 hour. **F**. APP-CTF levels are unchanged in 24 hour Thiamet-G treated hippocampal neurons. **G**. *O*-GlcNAc levels in primary hippocampal neurons are increased after 24 hours of treatment with Thiamet-G. (***indicates p <0.001,, paired two-tailed *t*-test) Error bars represent standard error of the mean (± S.E.M). N.S. Not Significant. For all panels, N =3.

In an effort to address the question of how *O*-GlcNAc might affect β-amyloid peptide formation we turned to using a well-established cell culture model used to evaluate molecular pathways influencing APP processing [35–38]. Using a cellular model enables us to ascertain the effects of OGA inhibition on β-amyloid peptide release independent of other factors that could obscure such effects in animals, such as β-amyloid peptide clearance and sequestration into plaques. The 20E2 cells we used are an established HEK cell line stably expressing APPSwe. The mutant APPSwe is processed by endogenous α, β and γ-secretase enzymes leading to abundant and readily detectable release of Aβ42 (Figure 7A) as compared to untransfected HEK cells (Figure 7B). We treated this 20E2 cell line with 100 μM Thiamet-G overnight and in the morning provided fresh media while maintaining 100 μM Thiamet-G. This dose of Thiamet-G selected for use in culture studies was chosen because we have previously found this dose to be well beyond the saturation point of increased *O*-GlcNAc levels in PC-12 cells [26] suggesting that *O*-GlcNAc levels are highly elevated, which should therefore stimulate possible effects within cells during these experiments. The cells and culture media were collected at 4, 8 and 24 hours after changing the media. While we were able to detect significant accumulation of Aβ42 over time as compared to non-transfected HEK cells, Thiamet-G did not exert any influence on release of Aβ42. We further verified that *O*-GlcNAc levels were increased at all time points in this experiment (Figure 7C). These data suggest that there are no differences in the rate of production or clearance of Aβ42. Nevertheless, because Aβ42 can also be cleared or degraded through various processes we considered a scenario wherein Aβ42 levels may appear unchanged by ELISA assay even though rates of production of Aβ42 could be different, perhaps due to compensatory degradation mechanisms. To address this possibility, we noted that Aβ42 production results from the cleavage by γ-secretase and therefore we assessed the levels of the γ-secretase substrate, the APP C-terminal fragment (APP-CTF). Here we also observed no difference in the levels of APP-CTF in 20E2 cells treated for 24 hours (Figure 7D). If changes in *O*-GlcNAc influenced the rates of Aβ42 production in these cells but such changes were obscured in our assays by compensating Aβ42 degradation, then we would expect APP-CTF levels would differ. The absence of any difference in APP-CTF levels, or the α:β ratio of CTF fragments, upon increased *O*-GlcNAc therefore provides further support that Aβ42 release and APP processing are unaffected by increased *O*-GlcNAc within this cell line.

As a further step to assess the effect of *O*-GlcNAc levels on APP processing in cells we used primary hippocampal neurons, which are a more physiologically relevant cell culture model. We assessed the levels of Aβ42 in the conditioned media from primary hippocampal neurons treated with Thiamet-G for 24 hours as well as the levels of APP-CTF in these same cells. Here, again, we observed no difference in either of these measures (Figure 7E, F) while *O*-GlcNAc levels were, as expected, dramatically increased in these neuronal cultures (Figure 7G). On the basis of these results, obtained using two distinct cell lines, 20E2 cells and primary neurons, we propose that increased *O*-GlcNAc does not affect the release of extracellular Aβ42 from cells. While a direct comparison is not possible between the cell culture studies and *in vivo* experiments, because the levels of Thiamet-G used here in cell culture likely exceed those reached in brain during dosing in water, these findings reasonably suggest there is also no effect on Aβ processing in the *in vivo* studies.

## Discussion

In the past number of years, the potential role of *O*-GlcNAc in AD has gradually gained more attention because of the fact that the *O*-GlcNAc modification is ultimately derived from cellular glucose. Through the action of the hexosamine biosynthetic pathway 2-3% of cellular glucose is converted into UDP-GlcNAc, which is the sugar donor substrate for OGT [39]. As a consequence, decreased glucose availability leads to decreased levels of UDP-GlcNAc and consequently lower protein *O*-GlcNAcylation. In this regard, it is notable that one of the early features of mild cognitive impairment and AD is impaired glucose utilization [40] in the brain which would result in decreased *O*-GlcNAc levels. Decreased expression of glucose transporters 1 (Glut1) and 3 (Glut3) have been found in AD brains and loss of function mutations in glucose transporter 1 (Glut1) associate with extent of AD pathology [41, 42]. Consistent with these observations, AD brain tissues contain less *O*-GlcNAc on cytosolic proteins [43]. Interestingly, another disease where glucose utilization is impaired, diabetes, is a major risk factor for AD [44]. Together, these ideas suggest that *O*-GlcNAc may play a protective function in the healthy brain and loss of *O*-GlcNAc in AD brain could compromise such protective mechanisms.

Until recently, the role of *O*-GlcNAc in AD focused exclusively on the *O*-GlcNAcylation of tau and its interplay with tau phosphorylation and tau aggregation [20, 26, 43, 45–47]. While analysis of the data from our long-term studies were underway a report on the role of *O*-GlcNAc on APP and β-amyloid was published, which made use of an earlier generation OGA inhibitor, NButGT [48], to increase *O*-GlcNAc levels in the 5xFAD mouse model [49]. NButGT is less potent (*K*_i_ = 600 nM) and selective (800-fold over functionally related β-*N*-acetylglucosaminidases) as well as less stable than Thiamet-G [26]. Thiamet-G therefore offers some advantages in long term studies in AD mouse models because it can be added to the drinking water of treated animals, thereby offering a simple dosing protocol. The 5xFAD mice studied by Kim *et. al* express five different early-onset AD mutations in human APP along with presenilin 1 but do not develop hyperphosphorylated tau or tau aggregates [50]. The 5xFAD mice are therefore a suitable model to study β-amyloid pathology independent of significant tau pathology. In these 5xFAD mice Kim *et al.* report that increased *O*-GlcNAc blocks memory impairment and results in fewer amyloid plaques. Their data are generally in good agreement with our data described here. We find prevention of memory impairment by increased *O*-GlcNAc is independent of tau phosphorylation as we observed no changes in tau phosphorylation. However, in order for this to be definitively proven Thiamet-G treatment of the parental Tg2576 line, which only displays amyloid pathology, would be necessary. Indeed, this would be a useful future endeavor to further clarify the role of *O*-GlcNAc on amyloid pathology.

Our data described here, with respect to tau pathology, are in general agreement with our previous studies using the single transgenic JNPL3 mice. In that work, we found that Thiamet-G did not block tau hyperphosphorylation but reduced the number of NFTs and the amount of sarkosyl insoluble tau [20]. In the TAPP mice studied here, we observe no changes in tau hyperphosphorylation but find a strong trend toward less sarkosyl insoluble tau in Thiamet-G treated animals. The fact that this finding did not reach statistical significance might be the result of a number of different factors. First, the TAPP mice are known to produce enhanced tau pathology as compared to similarly aged JNPL3 mice and this aggressive model may overwhelm or compromise the ability of the *O*-GlcNAc modification to impair tau aggregation [17]. Secondly, the JNPL3 mice display well characterized variable penetrance of the transgene leading to only ~50% of animals displaying hyperphosphorylated tau at advanced ages [51], which decreases the statistical power of the study.

In any event, the use of two OGA inhibitors and the concordance of results regarding β-amyloid pathology and cognitive impairment from our study in the dual pathology TAPP mice, along with the data collected from the single pathology 5xFAD mice studied by Kim *et al.*, provides compelling data that increased *O*-GlcNAc plays a pronounced role in hindering the development of β-amyloid pathology. The findings also collectively indicate that OGA inhibition is able to influence β-amyloid pathology either in the presence or absence of tau pathology.

The observation that 200 mkd Thiamet-G treatment was sufficient to reduce the number of amyloid plaques in the cortex of treated TAPP mice while leaving the amounts of Aβ40 or Aβ42 unchanged is interesting. The variation in the effects observed from these different doses may be due to varying levels of *O*-GlcNAc on different proteins between these two dose groups. Perhaps only at high doses of Thiamet-G, for extended time periods, are influences on β-amyloid peptide levels apparent. Lower doses of Thiamet-G may block only amyloid plaque deposition or influence its clearance. While the dosing of Thiamet-G in water, coupled with the lack of knowledge regarding the pharmacokinetics for Thiamet-G and the lack of detailed knowledge regarding the pharmacodynamic responsive of *O*-GlcNAc, make it difficult to know how specific *O*-GlcNAc levels influence these markers, we nevertheless see some evidence for a dose dependent response in *O*-GlcNAc levels to support this idea. Further supporting this idea, in the 20E2 cell model of APP processing, we do not observe a reduction in the amounts of Aβ42 released. Kim *et al.* suggest that increased *O*-GlcNAc levels could impair γ-secretase activity in CHO cells expressing APPSwe, leading to increased quantities of the membrane bound APP-CTF. However, the quantities of Aβ40 and Aβ42 were not determined, making direct comparisons with our study difficult. Another recent study found a non-selective OGA inhibitor increased *O*-GlcNAc levels in wild-type SH-SY5Y cells and this correlated with decreased Aβ40 release though Aβ42 levels were not evaluated [29]. Given the established off-target effects of PUGNAc and its ability to affect levels of cellular gangliosides as well as intracellular free oligosaccharides [48, 52, 53] these data should be interpreted with some caution. Given these observations, to further probe this question of the effects of *O*-GlcNAc levels on Aβ42 processing, we also evaluated levels of the γ-secretase substrate (APP-CTF) and product (Aβ42) in two separate models, the standard 20E2 cultured cell model noted above but also in primary hippocampal neurons since these are a more physiologically relevant model. In all these experiments, we observed no effect of Thiamet-G treatment on levels of Aβ42 released from cells, nor on levels of APP-CTF. This is a particularly interesting observation in the light of the fact that lower levels of Aβ42 are observed in Thiamet-G treated TAPP mice yet we observe no impairment of APP processing in cells. In this regard we hypothesize that increased *O*-GlcNAc levels could result in increased clearance of Aβ peptides or oligomers through various processes known to contribute to Aβ clearance, or perhaps by reducing neuroinflammation, which could lead to decreased tau and amyloid pathologies.

Nevertheless, these studies, combined with the literature noted just above, collectively reveal there is a need for further research to define the possible roles *O*-GlcNAc might play in APP processing. The clear effect of OGA inhibition on amyloid pathology in two different models using related, yet different, compounds also collectively provides a strong rationale for research studying how *O*-GlcNAc might influence plaque assembly and β-amyloid clearance. Such results could help in a more immediate way to define novel therapeutic avenues to address the growing problem of AD associated with aging populations.

## Conclusion

Here we have shown that Thiamet-G treatment of TAPP mice increases *O*-GlcNAc levels in the brain and completely prevents cognitive decline. Further, we have shown that this blockade in cognitive decline is not likely due to changes in tau hyperphosphorylation or tau aggregation but rather due to alteration in the release or clearance of β-amyloid peptides, which are reflected in the number of amyloid aggregates and plaques. Using a cellular model of APP processing we have shown that Thiamet-G does not impair release of β-amyloid peptides from APP and thus likely does not directly regulate APP processing, suggesting that *O*-GlcNAc probably acts at multiple other points in the β-amyloid pathological cascade. Nevertheless, OGA inhibition has positive effects on β-amyloid pathology in TAPP mice that develop synergistic tau and β-amyloid pathologies as shown here. This observation is consistent with observations made by Kim *et al.* in 5xFAD mice [49]. Because these mice display only amyloid pathology, the concordance of these results in different models strongly supports the hypothesis that *O*-GlcNAc exerts positive effects on β-amyloid pathology whether tau pathology is present or not, making the molecular mechanisms by which *O*-GlcNAc acts a topic of future interest. Finally, the ability of *O*-GlcNAc to influence both tau pathology, as we have previously shown [20, 46] and more recently others [54, 55], as well as β-amyloid pathology, makes OGA a potential therapeutic target of considerable interest since OGA inhibitors may be able to protect against both pathologies and serve as a monotherapy for altering disease progression in AD.

## Methods

### Animals

All animal studies were approved by the Simon Fraser University Animal Care Committee. 60 female double transgenic APPSwe-Tau (TAPP) mice were purchased and received from Taconic Farms at 9-12 weeks of age. 10 age-matched wild-type control animals were also obtained from Taconic Farms. The animals were allowed to acclimatize to the facility for one week after which dosing with OGA inhibitor was started. Thiamet-G was delivered to the treatment groups (n = 20) by including the compound in the drinking water such that each animal received either 200 or 500 mg/kg/day (mkd) based on an average water consumption of 4 mL/day/animal. Control TAPP mice (n = 20) and wild-type (n = 10) received free access to regular drinking water which averaged 4 mL/day. Following the completion of the study when mice were aged 44 to 47 weeks, both TAPP and wild-type mice were euthanized using CO_2_ and perfused transcardially with 15 mL of PBS (pH 7.4). Brains were dissected from mice immediately after they were euthanized in order to minimize post-mortem delay. Each brain was then divided along the midline to separate the left and right hemispheres. For soluble β-amyloid ELISAs, one hemisphere of brain was further microdissected into cortex, hippocampus, cerebellum and brainstem, quickly frozen in liquid nitrogen, and stored at –80°C until required. For amyloid plaque immunohistochemistry studies, the other hemisphere of the brain was post-fixed in 4% (w/v) paraformaldehyde (PFA, pH 7.4) for 24 h and then transferred to 20% (w/v) sucrose overnight for cryoprotection after which the tissue was embedded in optimal cutting temperature (OCT) embedding medium (Sakura Finetek USA Inc), and finally sectioned in the sagittal plane (30 μm) using a Leica cryostat.

### Behavioral testing

All mice were between 30-32 weeks old at the beginning of Morris water maze (MWM) testing. For five consecutive days prior to testing, the mice were removed from their cage, placed in an open cage on a table similar to the one being used to hold the animals during the testing stage. The animals were allowed to roam freely for 5 minutes and were gently handled as they would be during the testing to reduce any stress from these procedures [56]. This pre-test handling was done in the colony room. On the day of testing, the mice were removed from the colony room and taken to the testing room.

A 150 cm diameter pool was centered within a rectangular room measuring 470 cm × 400 cm, with a video camera (Sony DCR-SR85) centered overhead; behavior in the pool was tracked and quantified from the video feed using AnyMaze software (Stoelting). The walls of the room were affixed with various geometric symbols. The pool was filled with water at 23 ± 1°C and made opaque with non-toxic acrylic white paint. For analysis purposes, the pool was divided by the software into 4 equal imaginary quadrants, arbitrarily identified as northwest (NW), northeast (NE), southwest (SW) and southeast (SE). A hidden escape platform, approximately 25 cm^2^ and 2.5 cm below the water surface was randomly placed in the middle of the SE quadrant, and left in that location for the entirety of the testing period. For other analyses (described below), the maze was divided into 3 imaginary concentric rings: an inner ring, a middle ring, which contained the platform, and an outer ring along the wall.

Each animal was tested four times per day for five days. The animals were released into the pool from each of 4 starting locations every day in a pattern that was randomly determined prior to testing. For every trial, the animal was placed in the pool facing the wall. Animals were then allowed 90 seconds to find the platform. If they were unable to find the platform in that time, they were guided to it by hand. After allowing each animal to remain upon the platform for 15 seconds, they were removed. A minimum of 5 minutes elapsed between trials, during which time the animal was placed on an elevated platform in the testing room. A heat lamp was affixed above the platform. All testing started at noon, and the order in which the animals were tested was randomly changed, to prevent any time of day effects. For each trial, data was collected for total time spent in each quadrant or ring, distance covered, and swim speed. Time spent in the outer ring (thigmotaxis) was analyzed as a measure of anxiety in the maze.

One week following the final test of the acquisition phase, animals were returned to the MWM for one final (probe) test, with all animals starting from the same pre-determined starting location. The platform was removed from the water during this trial, however, and the amount of time spent over the previous location of the platform was measured as an indicator of long-term memory formation.

### Tissue culture

20E2 cells are a stable Human embryonic kidney (HEK) cell line which expresses the Swedish mutation (K670N/M671L) in the 695 amino acid isoform of APP [37]. 20E2 cells were kindly provided by Dr. Weihong Song (University of British Columbia). Cells were cultured in Dulbecco’s modified Eagle’s medium (DMEM) containing high glucose and 1.5 g/L sodium bicarbonate supplemented with 10% fetal bovine serum (FBS, Gibco). Cells were maintained in a humidified 37°C incubator with a 5% CO_2_ atmosphere. For the time course of β-amyloid production, 20E2 cells were plated onto 6 well plates. Thiamet-G was added 16 hours prior to the start of the experiment and upon initiation of the experiment the media was changed and Thiamet-G was maintained in the media to maintain continuous exposure. This is to ensure that *O*-GlcNAc levels were elevated prior to starting the experiment and maintained during the entire experiment. After completion of the experiment, the media was collected and the cells were removed from the plates by scraping. Cells were then pelleted by centrifugation (800 × g) for 5 min, gently washed once with PBS, and lysed using 1% SDS in PBS and heated at 95°C for 10 min. The cell lysates and the media samples were then either used immediately or aliquoted and stored at –80°C until required. The tissue culture media was then assayed for β-amyloid content by using the commercial ELISA assays described below.

Rat primary hippocampal neurons were prepared exactly as described previously [57] and cultured in primary neuron growth media (PNGM; Lonza). The astrocyte feeder layer for the neuronal co-culture was generated using neural progenitor cells as described [58]. A 50% volume media replacement was performed before neurons were treated with vehicle or 100 μM Thiamet-G for 24 hrs.

### Immunohistochemistry

Free-floating brain sections were rinsed with PBS (pH 7.4) three times for 45 min first, then permeabilized with PBS containing 0.3% Triton X-100 (PBST) for 30 min. After blocking with 10% normal goat serum (NGS) and 2.5% BSA in PBST for 60 min, sections were incubated with mouse IgG monoclonal anti-β-amyloid antibody (6E10) at 4°C overnight, which reacts to human Aβ peptide (1-16 aa) and was generated in-house at the NYIBR and used at a dilution of 1:1000. After washing three times with PBS for 45 min, sections were incubated with Alexa488 goat anti mouse IgG (Invitrogen) secondary antibody at 1:1000 for 90 min. After 45 min washing, the sections were mounted on pre-coated slides (Adhesion superfrost plus, Brain Research Laboratories, Newton, MA), and cover-slipped with Vectashield MountingMedium (H-1000, Vector Laboratories). Sections examined in parallel but without being exposed to primary antibody served as experimental controls. The stained sections were examined using a Nikon C1 confocal system equipped with Nikon 90i fluorescent microscope. The images were acquired by the Nikon digital camera and Nikon C1 imaging system.

### Aβ40 and Aβ42 ELISAs

Commercially available enzyme linked immunosorbant assay (ELISA) kits for amyloidogenic β-amyloid 1-40 and 1-42 (Aβ40 and Aβ42) (Invitrogen) were used to determine the total human Aβ40 and Aβ42 levels in the brain homogenates. Aβ42 was detected in neuronal cell culture media exactly as described by the manufacturer’s instruction using an ELISA kit from Wako (Human/Rat β-Amyloid(42)). Protein concentrations of the brain homogenates were determined using the BCA method (Pierce) and the Aβ40 and Aβ42 concentrations were expressed as ng Aβ40 or Aβ42/g of protein in the homogenate.

### Quantitative immunohistochemistry of amyloid plaques (APs)

One sagittal section per mouse was immuno-stained with 6E10 which recognizes amino acids 1-16 of β-amyloid. Immuno-positive APs were counted manually and expressed as the number of APs/section.

### Immunohistochemical staining

Frozen mouse brain sections (50 μm) stored in DeOlomos buffer at -20°C were washed with TBS and treated with 1% H_2_O_2_ and 50% methanol for 30 min to inactivate endogenous peroxidase. After being blocked with 5% normal goat serum in TBS for 1 hour, sections were incubated with the appropriate primary antibodies (see Table 1), followed by biotin-labeled secondary antibodies (Vector Laboratories), HRP-labeled avidin-biotin complex (Vector Laboratories), and the substrate DAB (3,3'-Diaminobenzidine, Sigma). The specimens were observed under a microscope (Nikon) and images captured using a digital camera DS-L2 (Nikon).

**Table 1:** **Antibodies used in this study**

Antibody	Epitope	2 ^nd^ Ab species	Dilution	Supplier
RL2	*O*-GlcNAc	Mouse	1:1000	Abcam
CTD110.6	*O*-GlcNAc	Mouse	1:1000	Covance
pS199	Tau-pS199	Rabbit	1:1000	Biosource
pS396	Tau-pS396	Rabbit	1:1000	Biosource
pT212	Tau-pT212	Rabbit	1:1000	Biosource
AT8	Tau-pS202/pT205	Mouse	1:1000	Thermo
AT180	Tau-pT231	Mouse	1:1000	Thermo
PHF-1	Tau-pS396/pS404	Mouse	1:1000	Dr. Peter Davies
12E8	Tau-pS262/pS356	Mouse	1:1000	Thermo
6E10	Amino acids 1-16 of β-amyloid	Mouse	1:1000	In-house at NYIBR

### Antibodies

All antibodies used in this study are described in the Table 1.

### Quantitative image analysis

Phosphorylated tau was visualized in sagittal brain sections from 6-9 mice each group by IHC staining. Using the identical microscope and camera settings, at least four digital images per sample were taken to reflect the overall staining in the pons region of brain. For the cortex and hippocampus, images at 20X were used. Only one image for hippocampus and one for frontal cortex were taken. All images were analyzed using the commercially available software program Image-Pro Plus version 4.0 for Windows (Media Cybernetics, Silver Spring, MD). The total immuno-intensity of the selected immuno-positive area was divided by the area size, and the values relative to that of the 0 mkd TAPP group are presented in the graphs. The proposed method allowed numerical analysis of the immunostaining intensity. The data from each group was normalized by the 0 mkd TAPP group.

### Statistics analysis

For the MWM testing repeated measures analysis of variance (RM-ANOVA) was used to examine the effects of Thiamet-G on the latency of mice to complete the maze, their distance traveled, their speed of movement, and their time in each of the quadrants, with Tukey’s *post-hoc* to determine group differences where appropriate. The probe trial was analyzed using a one-way ANOVA with Tukey’s *post-hoc* tests. For all other data two-tailed unpaired student’s t-tests were used except for the sarkosyl insoluble tau data where we had data from previous studies to inform the predicted direction of change thus allowing the use of a one-tailed test in this case.

### Western blotting

Brain tissue was homogenized at 4°C in 9 volumes of buffer (Buffer H) containing (50 mM Tris-HCl (pH 7.4), 20 μM UDP, 0.5 μM PUGNAc, 2 mM sodium othovanadate, 0.1 M NaF, 1.0 mM EGTA, 0.5 mM AEBSF, 2.0 μg/ml aprotinin, 10 μg/ml Leupeptin, 2.0 μg/ml pepstatin A) and the protein concentrations of the homogenates were determined using the BCA method (Pierce). The levels of global *O*-GlcNAcylation (CTD110.6 and RL2) and tau phosphorylation at various sites were determined by Western blot analysis using the antibodies listed in Table 1. Primary neurons were lysed in RIPA buffer containing Complete Protease Inhibitor Cocktail (Roche). Samples (10 μg) were resolved on 16% SDS-polyacrylamide gels and transferred to nitrocellulose membranes for blotting of APP-CTF. 30 μg of protein lysates was loaded per lane of a 4-20% SDS-polyacrylamide gel for blotting of *O*-GlcNAcylation (CTD110.6). Membranes were incubated with the following primary antibodies overnight at 4°C: anti-APP-CTF (1:2500, Abcam), anti-*O*-GlcNAc antibody (CTD110.6, 1:3000, Covance) and anti-actin (1:1000; Li-Cor). Quantitative analysis of immunoreactivity was carried out using images obtained by Li-Cor fluorescence and quantified using *ImageJ*. Quantitation of the lysate concentrations was performed using the BioRad QuickStart Bradford assay for both neurons and the 20E2 cells and levels of proteins established as a ratio of protein to actin.

### Sarkosyl extraction

Whole tissue homogenates described above were first centrifuged at 10,000 × g in an Eppendorf 5417C centrifuge for 20 min. Supernatants were adjusted to have a final concentration of 1% *N*-laurylsacrosinate (Sarkosyl, Sigma) and were incubated at 37°C for one hour with shaking. Samples were then centrifuged at 100,000 × g in a Beckman TLA-45 rotor in a Beckman TL-100 centrifuge at 4°C for 45 min. The supernatant was removed and the sarkosyl insoluble pellet was resuspended in SDS-PAGE loading buffer.

## Abbreviations

NFTs: Neurofibrillary tangles
AD: Alzheimer Disease
*O-*GlcNAc: *O*-linked N-Acetylglucosamine
APP: Amyloid precursor protein
OGA: *O*-GlcNAcase
FTDP-17: Frontal temporal dementia linked to chromosome-17
APPSwe: Swedish APP mutation
MWM: Morris water maze
ELISA: Enzyme linked immunosorbant assay
AP: Amyloid plaque
HEK: Human embryonic kidney.
